#  Correction

**DOI:** 10.1111/cas.15689

**Published:** 2023-03-16

In an article^1^ titled “Long noncoding RNA plasmacytoma variant translocation 1 promotes progression of colorectal cancer by sponging microRNA‐152‐3p and regulating E2F3/MAPK8 signaling” by Xin Liu, Lei Li, Jing Bai, Liang Li, Jianghe Fan, Zexian Fu, Jianxia Liu, the Conflict of Interest statement is missing.

The correct statement is shown below:

“There are no conflict of interests in this research.”

The authors apologize for the error.
